# Lean mass, muscle strength, and physical function in a diverse population of men: a population-based cross-sectional study

**DOI:** 10.1186/1471-2458-10-508

**Published:** 2010-08-21

**Authors:** Andre B Araujo, Gretchen R Chiu, Varant Kupelian, Susan A Hall, Rachel E Williams, Richard V Clark, John B McKinlay

**Affiliations:** 1 New England Research Institutes, Inc., Watertown, MA, USA; 2 GlaxoSmithKline, Collegeville, PA, USA; 3 GlaxoSmithKline Research and Development, Research Triangle Park, NC, USA

## Abstract

**Background:**

Age-related declines in lean body mass appear to be more rapid in men than in women but our understanding of muscle mass and function among different subgroups of men and their changes with age is quite limited. The objective of this analysis is to examine racial/ethnic differences and racial/ethnic group-specific cross-sectional age differences in measures of muscle mass, muscle strength, and physical function among men.

**Methods:**

Data were obtained from the Boston Area Community Health/Bone (BACH/Bone) Survey, a population-based, cross-sectional, observational survey. Subjects included 1,157 black, Hispanic, and white randomly-selected Boston men ages 30-79 y. Lean mass was assessed by dual-energy x-ray absorptiometry. Upper extremity (grip) strength was assessed with a hand dynamometer and lower extremity physical function was derived from walk and chair stand tests. Upper extremity strength and lower extremity physical function were also indexed by lean mass and lean mass was indexed by the square of height.

**Results:**

Mean age of the sample was 47.5 y. Substantial cross-sectional age differences in grip strength and physical function were consistent across race/ethnicity. Racial/ethnic differences, with and without adjustment for covariates, were evident in all outcomes except grip strength. Racial differences in lean mass did not translate into parallel differences in physical function. For instance, multivariate modeling (with adjustments for age, height, fat mass, self-rated health and physical activity) indicated that whereas total body lean mass was 2.43 kg (approximately 5%) higher in black compared with white men, black men had a physical function score that was approximately 20% lower than white men.

**Conclusions:**

In spite of lower levels of lean mass, the higher levels of physical function observed among white compared with non-white men in this study appear to be broadly consistent with known racial/ethnic differences in outcomes.

## Background

The aging process is accompanied by substantial changes in body composition, such as increases in fat mass [1] and loss of lean body mass [2]. The age-related decline in lean body mass that affects functional capacity, referred to as sarcopenia, [3] appears to be more rapid in men than in women [4,5]. Nonetheless, our understanding of muscle mass and function among different population subgroups of men and their changes with age is quite limited. For instance, relatively little is known about potential racial/ethnic differences among men. This is important since loss of muscle is strongly linked with disability, [6] which has further consequences for outcomes which are known to vary with race/ethnicity, including falls, osteoporotic fracture, quality of life, mortality, and health care expenditures [7-15]. Given the aging of the worldwide population, the public health and economic consequences of loss of muscle function will only increase unless strategies are developed to reduce its occurrence.

The objective of this analysis is to examine racial/ethnic differences and racial/ethnic group-specific cross-sectional age differences in measures of muscle mass, muscle strength, and physical function in a racially/ethnically diverse population-based sample of men from Boston.

## Methods

### Study sample

Data were obtained from men enrolled in the Boston Area Community Health/Bone (BACH/Bone) Survey, which is a cross-sectional observational study of skeletal health and related outcomes in 1,219 (of 1,877 eligible, 65% response rate) randomly selected black, Hispanic, and white male Boston, MA residents aged 30 to 79 y [16]. Persons of other racial/ethnic backgrounds were not enrolled. BACH/Bone Survey subjects were a subset of 2,301 men previously enrolled in the parent Boston Area Community Health (BACH) Survey; full details of the BACH survey have been published previously [17]. Study protocols were approved by Institutional Review Boards at New England Research Institutes (NERI) and Boston University School of Medicine (BUSM). All subjects gave written informed consent separately for participation in each study.

### Data collection

Trained staff at NERI and the BUSM's General Clinical Research Center (GCRC) conducted interviews and measurements for BACH and BACH/Bone, respectively. Data collection for BACH generally occurred in subjects' homes while data collection in BACH/Bone occurred at the BUSM GCRC. Age and self-rated health were obtained by self-report. Physical activity level was measured using the Physical Activity for the Elderly (PASE) scale [18]. Frequency and duration of leisure activities, paid or unpaid work (hours/week), and housework and similar duties (yes/no) over the past week were recorded for each subject. The PASE score was computed by multiplying the amount of time spent in each activity (hours/week) or participation (yes/no) in each activity by empirical item weights (derived from regressions of component scores developed from a 3-day physical activity monitor, 3-day physical activity diary, and a global self-report of physical activity on responses to the PASE in a community-dwelling sample of 277 older adults [18]) and summing over all activities. Measurements of subjects' height and weight were obtained using a stadiometer (Seca Corp., Hanover, MD) and digital scale (Tanita, Arlington Heights, IL), respectively. Body mass index (BMI) was calculated from by dividing measured weight (kg) by the square of measured height (m^2^).

### Measures of body composition

Measurements of lean mass and fat mass were obtained from whole body dual energy x-ray absorptiometry (DXA) scans using a QDR 4500 W densitometer (Hologic, Inc., Waltham, MA) located at the BUSM GCRC. All mass quantities reported here exclude the head. Lean mass was calculated by subtracting bone mineral mass from non-fat mass. The DXA system was monitored weekly for drift.

### Measures of strength

BACH/Bone has measures of subjects' upper and lower extremity strength/physical function. Upper extremity strength was assessed by hand grip strength. This was measured using a Jamar Hydraulic Hand Dynamometer (Sammons Preston, Bolingbrook, IL), which measures isometric grip force. Subjects were instructed to exert maximum effort for three seconds during two trials, each separated by a 1-min rest. The maximum result was used for analysis. Lower extremity strength was assessed by a chair stand test (time to stand up and sit down 5 times) and a walking test (time to walk 50 ft) [19]. Following a previous study, [19] we created a lower extremity composite physical function variable. Those who could not complete the test were assigned a score of 0. Those completing the walk and chair stand tests were assigned scores of 1-4, corresponding to the quartiles (derived from our population) of speeds in completing each task, with the fastest speeds scored 4. The cutpoints for walking speed were as follows: quartile 1, <1.19 m/s; quartile 2, 1.19-1.30 m/s; quartile 3, 1.31-1.40 m/s; quartile 4, ≥1.41 m/s. The cutpoints for chair stand speed were quartile 1, <0.314 stands/s; quartile 2, 0.314-0.360 stands/s; quartile 3, 0.361-0.430 stands/s; quartile 4, ≥0.431 stands/s. Only one subject was not able to complete the walk task, so we included that subject with those who were in the slowest quartile of walking speed and reassigned the walking speed quartiles to scores 0-3. The two items were summed to a final score with possible range of 0 to 7, with higher scores indicating better lower extremity function.

### Indexed outcomes

Outcomes were indexed by either regional lean mass or the square of height as appropriate. Lean mass was divided by the square of height in meters to yield the lean mass index (LMI). Additional measures of relative strength in the upper and lower extremities was estimated by dividing upper and lower extremity measures of strength/physical function by their corresponding regional measures of lean mass [20]. Specifically, maximum grip strength was divided by arms lean mass and the lower extremity composite physical function score was divided by legs lean mass.

### Analysis samples

Of the 1,219 men in BACH/Bone, 10 men did not have DXA scans performed. Of the remaining 1,209, we excluded 49 who were missing fat or lean mass and three men missing PASE. This left 1,157 men available as a base for analysis. From this base analysis sample, we used the maximum available data for each of the outcome measures: lean mass and lower extremity strength, N = 1,147; upper extremity strength, N = 970 (54 men were coded as missing due to dynamometer malfunction).

### Statistical methodology

Sampling weights were used to produce estimates for means and percentages that are representative of the black, Hispanic, and white male population in Boston, MA between the ages of 30 and 79 y. Sampling weights account for the design effect of over-sampling of particular age and racial and ethnic groups [21].

Exploratory graphical analysis was conducted using locally weighted linear regression (LOESS) models where non-linear functions are fit to subsets of the data using weighted least squares [22]. Line graphs showing cross-sectional age differences in the main outcomes by race/ethnicity are presented.

Three multivariate linear regression models were constructed for each of the outcome variables: (1) Model 1: adjusted for age, race/ethnicity, and height. (2) Model 2: all variables in Model 1 plus potential confounding influences (fat mass, self-rated health status and physical activity). Smoking and a count of 6 major medical comorbidities were also examined but they made no significant contribution to any of the models. (3) Model 3: all variables in Model 2 plus grip strength, the composite physical function score, or lean mass (for non-indexed outcomes) or grip strength/arms lean mass, composite physical function score/legs lean mass, or lean mass index (for indexed outcomes). For instance, Model 3 with lean mass as the outcome would have included all factors in Model 2, plus grip strength and the composite physical function score. This third set of models was constructed in order to examine which variables differ most between the racial/ethnic groups in the presence of other muscle function variables of interest. Race/ethnicity was placed in each model as a categorical variable. Regression coefficients and 95% confidence intervals (CI) for black and Hispanic men (using white men as the reference category) were presented. Associations were considered statistically significant if null hypotheses could be rejected at the 0.05 level (two-sided). All statistical modeling was conducted using SUDAAN software (Research Triangle Institute, Research Triangle Park, NC).

## Results

The mean age of the sample was 48 y. Descriptive statistics for several variables of interest can be found by race/ethnicity in Table 1. Lean mass and grip strength was similar in black and white men, while white men had a 25% higher average composite physical function score. When these measures were indexed by lean mass black/white differences were accentuated (grip/lean mass: 10% higher among white men; physical function/lean mass: 29% higher among white men). LMI was 5% higher in black compared with white men. White men also had higher lean mass, grip strength, and composite physical function score when compared to Hispanic men, but these differences were reduced to <10% when indexed for height or lean mass.

**Table 1:** Descriptive statistics^a ^by race/ethnicity (N = 1,157).

	Black		Hispanic		White		
	
Variable	Mean	(SD)	Mean	(SD)	Mean	(SD)	Comparison^b^
**Lean Mass, kg**	56.35	(8.83)	51.82	(7.24)	55.43	(7.14)	H < B, W
**Arm Lean Mass, kg**	7.84	(1.58)	7.05	(1.28)	7.05	(1.18)	H, W < B
**Leg Lean Mass, kg**	20.21	(3.58)	18.06	(2.83)	19.40	(2.76)	H < W < B
**Grip Strength, kg**	40.83	(12.99)	37.59	(8.60)	40.24	(11.48)	H < B, W
**Composite Physical Function Score**	3.45	(1.97)	3.66	(1.84)	4.30	(1.99)	B, H < W
**Lean Mass Index, kg/m^2^**	18.41	(2.34)	17.99	(2.21)	17.59	(2.02)	H, W < B
**Grip Strength/Arms Lean Mass**	5.21	(1.38)	5.40	(1.30)	5.71	(1.50)	B, H < W
**Composite Physical Function Score/Legs Lean Mass**	0.17	(0.10)	0.21	(0.11)	0.22	(0.11)	B < H, W
**Height, cm**	174.72	(7.31)	169.63	(6.14)	177.46	(6.93)	H < B < W
**Weight, kg**	87.78	(16.84)	81.54	(14.27)	89.04	(14.73)	H < B, W
**BMI, kg/m^2^**	28.72	(5.07)	28.33	(4.72)	28.27	(4.44)	B, H, W
**Fat Mass, kg**	20.57	(9.03)	19.78	(7.37)	23.10	(8.47)	B, H < W
**Percent Fat Mass**	25.74	(7.60)	26.86	(6.09)	28.65	(6.50)	B, H < W

^a ^All estimates weighted according to Sampling Design (see Methods)

^b ^Pairwise comparison, p < .05. B = Black, H = Hispanic, W = White

**Figure 1:**
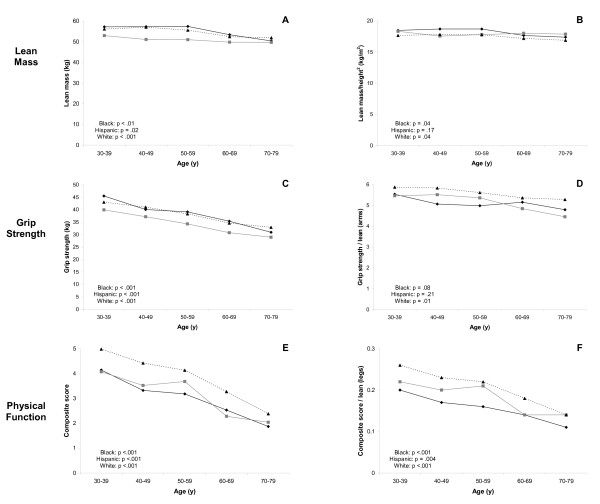
**Cross-sectional age differences in outcomes by race/ethnicity**. Panel A: Lean mass; Panel B: Lean mass index; Panel C: Grip strength; Panel D: Grip strength/arms LM; Panel E: Composite physical function score; and Panel F: Composite physical function score/legs LM. Black men: Black diamonds with solid black line; Hispanic men: Grey squares with solid grey line; and White men: Black triangles with dashed black line. P-values test the null hypothesis of no age difference in outcome within each racial/ethnic group.

Figure 1 shows that strong age differences in grip strength (all p < .001), composite physical function score (all p < .001), and outcomes indexed by region-specific lean mass were consistent across race/ethnicity. Age differences in lean mass and LMI were less pronounced but also did not appear to differ appreciably by race/ethnicity. The absence of racial/ethnic differences in age trends in the outcomes was further verified with statistical tests for effect modification by race/ethnicity, none of which were significant (all p > .3). Figure 1 also shows that age differences in grip strength and in lower extremity physical function were more pronounced than their corresponding indicators which were indexed by region-specific lean mass; such was not the case when comparing age differences in lean mass and LMI. For instance, compared to men in their 30 s, men in their 70 s had 25% lower grip strength. In contrast, grip strength/arms lean mass was 11% lower among men in their 70 s. A similar pattern was evident in the lower extremity.

Exploratory scatter plots with LOESS curves were examined for lean mass, strength, and physical function vs. fat mass or weight (data not shown). Overall, fat mass appeared to be most strongly associated with the outcomes of interest. Also, although a slight curvature was observed (e.g., the association of fat mass with grip strength became less pronounced with increasing fat mass), formal statistical testing indicated that quadratic terms for fat mass contributed little to the model.

Results from the multiple linear regression models are depicted in Table 2. Grip strength was not associated with race/ethnicity independent of age, height, fat mass, self-rated health, and physical activity (p = .15). In contrast, grip strength/arms lean mass differed significantly by race/ethnicity, with higher estimates observed among white compared to black and Hispanic subjects (p < .01). However, further adjustment for composite physical function score and LMI confounded this association (p = .15). Values for the composite physical function score and composite physical function score/legs lean mass were higher in white men, even in the presence of confounders (both p < .001) and when further adjusted for grip strength and lean mass or grip strength/arms lean mass and LMI (both p < .001). Lean mass and LMI were higher in black (p < .001) and slightly higher in Hispanic (p = .06) men compared with white men when adjusted for all confounding influences. It is notable that the regression coefficients in models of lean mass and LMI increased with the addition of control variables to each model, indicating negative confounding on the part of these other variables.

**Table 2:** Results^a ^of multiple regression modeling.

	Regression coefficient *vs*. White (95% CI)		
	
	Black	Hispanic	Model R^2^
Lean Mass (kg)			
Model 1^c^	2.43 (1.12, 3.73)***	0.19 (-1.23, 1.60)	0.3094
Model 2^d^	3.41 (2.39, 4.44)***	1.06 (-0.05, 2.16)	0.5546
Model 3^e^	3.78 (2.78, 4.77)***	1.63 (0.40, 2.86)**	0.6087
			
Grip Strength (kg) ^b^			
Model 1^c^	1.60 (-1.67, 4.86)	-1.06 (-3.92, 1.80)	0.1306
Model 2^d^	1.75 (-1.57, 5.07)	-0.98 (-4.08, 2.11)	0.1620
Model 3^e^	0.87 (-2.07, 3.81)	-0.90 (-3.64, 1.85)	0.2781
			
Composite Physical Function Score			
Model 1^c^	-0.89 (-1.22, -0.56)***	-0.94 (-1.41, -0.47)***	0.1600
Model 2^d^	-0.85 (-1.20, -0.49)***	-0.87 (-1.38, -0.35)***	0.2323
Model 3^e^	-1.04 (-1.40, -0.68)***	-0.79 (-1.25, -0.33)***	0.2671
			
			
**Lean Mass Index (kg/m^2^) ^g^**			
Model 1^c^	0.82 (0.40, 1.23)***	0.23 (-0.21, 0.67)	0.0411
Model 2^d^	1.18 (0.86, 1.50)	0.69 (0.33, 1.04)***	0.3630
Model 3^f^	1.25 (0.89, 1.61)***	0.70 (0.30, 1.10)***	0.3914
			
**Grip Strength/Arms Lean Mass**			
Model 1^c^	-0.54 (-0.91, -0.18)**	-0.50 (-0.87, -0.12)**	0.0450
Model 2^d^	-0.57 (-0.95, -0.20)**	-0.52 (-0.92, -0.13)**	0.0686
Model 3^f^	-0.23 (-0.59, 0.14)	-0.36 (-0.73, 0.01)	0.1396
			
**Composite Physical Function Score/Legs Lean Mass**			
Model 1^c^	-0.058 (-0.077, -0.040)***	-0.046 (-0.074, -0.019)**	0.1499
Model 2^d^	-0.060 (-0.079, -0.040)***	-0.046 (-0.075, -0.016)**	0.2567
Model 3^f^	-0.052 (-0.071, -0.0327)***	-0.0346 (-0.059, -0.010)**	0.2783

^a ^All estimates weighted according to Sampling Design (see Methods)

^b ^Models including grip strength or grip strength/LM have N = 970. Other models, N = 1,147.

^c ^Model 1: Controlled for age and height

^d ^Model 2: Controlled for age, height, fat mass, self-rated health and physical activity

^e ^Model 3: Controlled for age, height, fat mass, self-rated health and physical activity, plus maximum grip strength, the composite physical function score, or lean mass

^f ^Model 3: Controlled for age, height, fat mass, self-rated health and physical activity, plus maximum grip strength/arms LM, composite physical function score/legs LM, or lean mass index

^g ^Lean mass index models were not adjusted for height since height^2 ^is the denominator of the outcome

* p < .05

** p < .01

*** p < .001

## Discussion

In this population-based survey of diverse men, we find evidence of age and racial/ethnic differences in measures of lean mass and LMI, as well as lower extremity strength and physical function. Overall, more pronounced age differences were observed in strength measures as compared to lean mass and relative measures of strength (i.e., outcomes relative to the amount of regional lean tissue). Observed racial/ethnic differences indicate higher lean mass among black and Hispanic compared with white men but surprisingly, lower levels of physical function among these black and Hispanic men.

Previous studies have shown that there are racial disparities in disability, with lower rates generally reported among whites [23,24]. Although studies of diverse populations of men are rare, the few data on age changes in muscle function in such populations show complexities similar to those reported in the current study. Consistent with our study, data from the Chicago Health and Aging Project indicate that black men and women have lower physical function compared with white men and women [25]. They also observed that this difference increases over time, particularly among women. Data from the Third National Health and Nutrition Examination Survey indicate that while black men exhibit elevated lean mass as compared to white men in young adulthood, they also show accelerated loss of lean mass after age 50 [26,27]. Data from the current study are similar insofar as black men have higher lean mass compared with white men, but age differences by race/ethnicity were not observed in the current study. In the Health, Aging, and Body Composition Study black subjects had greater appendicular lean mass and strength, but also lower muscle quality than whites [20]. The data reported herein are quite consistent with these results, insofar as we observed that white men had lower lean mass but greater physical function than non-white men. The reasons for this apparent contradiction are not immediately clear, but one could speculate that this could relate to differences in muscle quality, or the strength exerted for each unit of muscle, between racial/ethnic groups. Unfortunately, this study did not collect the data required to address this issue. Nonetheless, the finding that higher lean mass among non-white men does not translate into better grip strength or physical function is broadly consistent with general findings of worse health outcomes (morbidity, mortality) among non-white men. Finally, while these data do not necessarily rule out a role for muscle function as determinants of racial/ethnic differences in fracture risk, they emphasize the central role of racial/ethnic differences of bone strength [16,28,29].

Contributors to muscle strength and physical function are not well understood. Studies show that age-related loss of strength is greater than muscle mass, [30,31] as we observed in this study. This suggests that muscle quality may be reduced with aging, although as noted previously, this study did not have data to test this. If loss of muscle is the sole factor responsible for age declines or racial/ethnic group differences in muscle strength or physical function, then relative strength measures should not differ according to age or race/ethnicity. This explanation is not consistent with the observations in this study. Racial/ethnic differences in lower extremity physical function were observed even after indexing for lower extremity lean mass, as observed in other studies [20]. In analyses not shown, lean mass accounted for only a small portion of observed age differences in muscle strength, which is consistent with some studies [32-35] but not others [30,36-39]. These discrepancies could relate to the observation that age declines in measures of relative muscle strength may depend on how muscle mass is estimated and the study design [31].

Limitations to the current study should be acknowledged. First, the cross-sectional design of the study is problematic, limiting our ability give a true estimate of age trends. Second, the dynamometer used in this and many other epidemiologic studies does not measure maximal voluntary strength, which is a superior measure of muscle strength. An obvious limitation to the current study is the lack of data on specific endpoints of interest (e.g., fracture, disability, mortality), limiting our ability to examine whether observed differences in the muscle-related factors examined "explain" racial/ethnic differences in these endpoints. Finally, subjects were not asked to perform the tandem balance test, the third component of the short physical performance battery of Guralnik et al. [19] In addition, the cutpoints for the walk and chair stand test established by Guralnik et al. were not used in this report. This has been done previously with this measure by us [40] and others [41] and also for similar constructs [42,43]. Given the generally high levels of function of the population under study, the use of these established cutpoints induces a ceiling effect in this data set. This limitation is partially offset by the observation that the modified composite physical function score correlates well with theoretically related variables included in this analysis (age, self-rated health, and physical activity), and the appeal of the composite physical function score as a global measure of physical function.

## Conclusions

In conclusion, these data on racial/ethnic differences in lean mass and physical function, when considered as a whole, appear to be broadly consistent with known racial/ethnic differences in outcomes. Further exploration of why higher lean mass in non-white subjects do not appear to translate into higher strength and physical function is warranted. The observations reported herein could have implications for clinical trials as well as public health. These data raise interesting questions regarding the choice of endpoints in clinical trials given that our data on upper extremity strength suggest that the amount of muscle required to generate a given force may differ in important ways across individuals or groups of individuals. Our findings also have implications for public health. Racial/ethnic population subgroups are growing at varying rates, with non-white populations in the US growing more rapidly. For instance, the US Hispanic population represented approximately 12% of the population in 2000 and will represent 24% of the population in 2050. This means that the lower levels of physical function observed among black and Hispanic men, insofar as this may translate into higher rates of physical disability and its associated consequences, could result in increased health care costs as the age distribution of these population subgroups shifts towards an older age.

## Competing interests

A.B.A., G.R.C., V.K., S.A.H., and J.B.M. received funding for analysis and write-up of the current manuscript from GlaxoSmithKline. R.E.W. and R.V.C. are employees of, and have equity interest in, GlaxoSmithKline.

## Authors' contributions

All authors 1) made substantial contributions to conception and design, acquisition of data, or analysis and interpretation of data; 2) drafted the article or revised it critically for important intellectual content; and 3) provided final approval of the version submitted.

## Authors' Information

NA

## Pre-publication history

The pre-publication history for this paper can be accessed here:

http://www.biomedcentral.com/1471-2458/10/508/prepub
